# The National Medical Union (Official)

**Published:** 1914-04-04

**Authors:** 


### THE NATIONAL MEDICAL UNION (Official).

Offices: 346 Strand. Tel.: City 8444.

### RESOLUTIONS ON POLICY.

At the meeting of the Council on Saturday, March 21, the Chairman moved *seriatim* the resolutions of the various Associations. The first was:—That it be the policy of the National Medical Union:—

1. *To organise the medical profession to the highest possible degree of efficiency in defence of its freedom and protection of its economic interests.*

Dr. Ford Anderson proposed that this resolution should read, "*its traditional freedom.*"

2. *To render every service possible to non-panel practitioners in their unequal struggle against the difficulties brought about by acceptation by panel practitioners of service under the National Insurance Acts.* This was agreed to.

3. *To appoint a Parliamentary Committee whose duties shall include lobbying, formulating questions for Parliament, approaching possible candidates at elections, and doing everything possible to bring the policy of the Union before Parliament.*

Dr. Johnston was of opinion that this was a matter of constitution rather than of policy, and the proposal was made and agreed to that this matter should be left to the Constitution Committee with instructions from the Council to incorporate it in the draft constitution.

4. *That a Committee be appointed consisting of the London members of the Council, with such other London members of the Union as may be co-opted from time to time, with power to act at its discretion in all matters appertaining to the Metropolitan area.*

This also was relegated to the Constitution Committee for the same reason and with a similar instruction.

5. *To oppose any prospective legislation to include dependants of insured persons participating in the medical benefit of the Acts.*

This was agreed to, with the addition of the words "*under the present arrangements.*"

At this point Professor Russell moved that the resolution of Edinburgh in favour of drastic amendment of the Acts be taken together with the Wandsworth resolution on repeal of the medical benefit section. Professor Russell said that, representing Edinburgh, he was in favour of drastic amendment rather than repeal, as being less extreme and more likely to be acceptable to the public. Drastic amendment would mean repeal of clauses only. The pronouncement of the Union on this subject would affect the public estimation of its position. Repeal would be an impracticable position to take up, while drastic amendment would cover all that we want.

Mr. Wright supported this view, and was opposed to any action which seemed to call for the repeal of the Act altogether or would show that the Union was opposed to any form of insurance.

Dr. Greene pointed out that repeal of the medical benefit *section* might be regarded as drastic amendment of the *Acts*.

The Edinburgh resolution was then submitted to the meeting and agreed to—viz.

6. *That the policy of the National Medical Union be the drastic amendment of the National Insurance Acts in accordance with the principle of freedom on the part of the profession as laid down in its policy.*

The meeting then proceeded to consider the resolution in favour of repeal of the medical benefit section as part of the policy of the Union. In support of this resolution Dr. Greene said that the only practical position for the Union to take up was, in the first instance, to sweep away the present arrangements, to abolish the panels, and restore the patient to the sphere of common practice; and this whether or not any fresh scheme might be originated in the future. This would bring about a condition of affairs similar to what obtains at the present moment in Ireland, where there is no medical benefit, but the ground is covered by an extended Poor-Law system. Both political parties supported what was known as the contributory principle. Without abandoning this principle the Government, while still collecting the tax, could enable the approved societies to return it to insured persons in the form of pensions and other benefits. The effect of repeal of the medical benefit section on the friendly societies would be to restore them to a more satisfactory financial condition. Opposition would, therefore, not be met from that quarter. The industrial insurance societies were opposed to the present administration of medical benefit. They had never previously administered medical benefit and did not want to do so now. There would, therefore, be no opposition from them. Again, there was the fact that interested parties were to be found on both sides of the House as directors of insurance companies, or as otherwise involved in the conduct of insurance business. Thus members of both political parties had a common interest in the present Insurance Acts. He regarded repeal of the medical benefit section as an indispensable step in remedying the present panel system. Under this expansion of contract practice the profession was degraded, its ethics destroyed, and its independence stifled by lay control. The refusal to permit free choice by Insurance Committees accentuated the evil.

Dr. Carswell moved as an amendment that the words "or such drastic amendment as would bring these sections into harmony with the principles of freedom on the part of the profession and public as laid down in the policy of the Union" be added. He thought that this, while meeting Dr. Greene's points, would have less appearance of being a purely destructive policy. The medical profession was not opposed to insurance against sickness as an abstract principle. Dr. Sharman supported this amendment, as it had the appearance of being less extreme, and on being put to the meeting it was carried with four dissentients. The resolution reads as follows: 7. *Repeal of the medical benefit sections, or such drastic amendment of these sections as would bring them into harmony with the principles of freedom on the part of the profession and public as laid down in the policy of the Union.*

8. *To lay down the principle of unrestricted free choice of doctor in any form of National Insurance, and to insist upon the unrestricted right of insured persons to make their own arrangements in connection with the Insurance Acts*, 1911-13.

Dr. Ford Anderson proposed that the Hampstead resolution be taken instead. In the course of discussion on the Hampstead resolution it was pointed out that it was unnecessary and inexpedient to give recognition to any system of contract between patients and practitioners; and upon Dr. Sharman proposing that the words "*that no doctor attending such persons be required to sign any contract with the Commissioners or any committee under the Acts*" be added to the Wandsworth resolution, the latter was agreed to with that addition.

9. *To protest against the distribution of any unallotted medical benefit funds amongst panel doctors* until inquiry has been made amongst all the insured persons who have not selected a panel doctor, to ascertain how many of them (*a*) have been attended since the Act by non-panel doctors during illness, or (*b*) intend, if illness occurs, to be so attended. The sums due on account of medical benefit to all such persons should be placed in an "Own Arrangements" Fund and used for defraying, as far as such funds go, the medical expenses of these persons. With regard to this resolution it was agreed to omit everything after the word "doctors" in the third line.

10. To lay down the principle that payment of insurance premiums should be made collectively by the Insurance authority to associations of doctors, by whom the sum total should be divided on the basis of payment for work done.

The Chairman said that the subject of this resolution—namely, method of remuneration and control, together with the subject of free choice of doctor, lay at the root of the whole matter. He was of opinion that if a scale of minimum fees should be established and agreed upon by the whole medical profession it would go far to solve the difficulty. Dr. Carswell said that theoretically at least payments by sickness Insurance authorities should be in the form of capitation fees. It would be impossible to make payments on the basis of work done without exercising strict control, and this would mean irritating control by lay persons in technical matters, of which they were unfitted to judge. On the other hand, from the point of view of the doctors, payment for work done was the only system that was fair to individual doctors, and at the same time in the best interests of the patient. Given free choice of doctor, the solution of the problem seemed to lie in a combination of the two methods—namely, capitation payments on the part of Insurance authorities to corporations of doctors by whom individual medical men would be paid on the basis of work done. It would be an advantage under any such system that the patient should pay a small fraction of the fee. This would check malingering or valetudinarianism and make for economy. Dr. Morison said that this matter would require to be carefully considered in all its bearings, and he therefore advised that the Council should not make any pronouncement upon it at present, but that it should be relegated to a subcommittee to be carefully considered. Dr. Burgess said that it brought us back to the public medical service schemes which had been worked out prior to the coming into force of the Act. Dr. Prowse said that something of the kind had been tried at Manchester and Salford under the Insurance Acts and that it had proved unsatisfactory to all parties—medical men, Insurance Committees, and patients alike. Dr. Pinder said that the Insurance Acts had developed all the bad points in the profession. (A Voice: "Showed them up.") No, developed. It had lowered the standard of medical ethics. At Manchester over-attendance had been enormous and the drug bill double what it ought to be.

Dr. Johnston thought that medical opinion was divided on the subject of remuneration, some preferring capitation payment, others payment for work done, and that there was no unanimity. Dr. Heatherley said that in this connection the system of the National Deposit Friendly Society ought to be brought forward. Dr. Carswell, with reference to Dr. Prowse's remarks, said that at Manchester the medical profession had been actively organised before the questions involved in the National Insurance Act came before the profession. A joint committee of four or five divisions of the British Medical Association had become an influential executive in Manchester in connection with a proposal to appoint whole-time obstetric surgeons. In this matter their organisation was successful, and they were able to enforce a system of payment for work done in preference to the institution of whole-time appointments. Medical men at Manchester were therefore experienced organisers, and it was possibly due to this preliminary training that the medical profession in Manchester, which had at one time been so actively and strongly opposed to the Insurance Acts, adopted the pooling system, which was in some ways an illustration of the principles involved in the resolution. Although the Manchester system was said to be so unsatisfactory it was possible to regard it as a step in the right direction. One thing demonstrated was that by this system a clear view could be obtained of the amount of insurance premium which would be necessary for insured persons to pay in order to cover adequate attendance. If the premium were deficient, as it was in Manchester, the inevitable conclusion was either that the profession would have to reduce its services to a corresponding and inadequate extent or do charitable work.

Eventually Dr. Morison's proposal was agreed to—namely, that this resolution, together with a consideration of the systems of the National Deposit Friendly Society and the Scottish Clerks Association, be referred to a sub-committee for consideration and report.

**The remainder of the proceedings will appear next week.)**

